# Comparison of Patient Acceptance and Caregiver Satisfaction of Glass-Ionomer Cement vs. Silver Fluoride/Potassium Iodide Application to Manage Molar Incisor Hypomineralisation Hypersensitivity Immediately and After 12 Weeks

**DOI:** 10.3390/clinpract15020029

**Published:** 2025-01-31

**Authors:** Ramiar Karim, Walaa Ahmed, Mohamed Baider, Christian H. Splieth, Julian Schmoeckel

**Affiliations:** Department of Paediatric Dentistry, University of Greifswald, Walther-Rathenau-Straße 42a, 17489 Greifswald, Germany; walaa.ahmed@stud.uni-greifswald.de (W.A.); mohamed.baider@uni-greifswald.de (M.B.); splieth@uni-greifswald.de (C.H.S.); julian.schmoeckel@uni-greifswald.de (J.S.)

**Keywords:** hypersensitivity, molar incisor hypomineralisation, glass-ionomer cement, silver fluoride, Schiff score air sensitivity scale

## Abstract

**Aim:** To compare caregiver satisfaction and children’s acceptance of silver fluoride/potassium iodide (AgF + KI) treatment (Riva Star Aqua^®^, SDI Limited, Victoria, Australia) and glass-ionomer cement (GIC) application (Ionostar Plus + Easy Glaze, VOCO, Germany) in reducing hypersensitivity in permanent molars affected by molar incisor hypomineralisation (MIH) with the MIH treatment need index (MIH-TNI) 3 and 4 immediately after its application and after 12 weeks. **Materials and Methods:** This prospective, comparative, clinical study recruited schoolchildren with at least one hypersensitive MIH molar with a Schiff cold air sensitivity score (SCASS) of 2 and 3. Caregivers in both groups (AgF + KI and GIC + glaze) answered a questionnaire (5-Point Likert Scale) regarding the perception of the treatment immediately (15 min post application) and in the 12 weeks follow-up. Children’s behaviour during both applications was assessed using FBRS (Frankl Behaviour Rating Scale). **Results:** A total number of 47 children (n = 22 for AgF/KI and n = 25 for GIC) with a mean age of 8.6 ± 1.42 were recruited. A high proportion of the children in both arms (n = 40 out of 44; 90.1%) reported a reduction in hypersensitivity in the last 12 weeks. On average, children (n = 39; FBRS ≥ 3) in both groups showed positive behaviour, with a significantly more definitely positive behaviour in the GIC group (*p* < 0.05, independent student *t*-test). Caregiver satisfaction with both study procedures was high after immediate assessment (n = 19 out of 22, 86.4% for AgF/KI and n = 19 out of 25, 76.0% for GIC application) and in 12 weeks of follow-up (n = 17 out of 20, 85.0% for AgF/KI and n = 22 out of 24, 91.6% for GIC application). However, the taste AgF/KI is more frequently considered not acceptable for the child (n = 10; 45%) than smell (n = 2; 9%). Interestingly, there was a statistically significant difference in caregivers’ preference toward alternative desensitisation treatment (tooth restoration coverage, desensitisation paste, stainless steel crown and fluoride varnish) in both treatment groups (*p* < 0.05, Mann–Whitney U test). **Conclusions:** Both GIC and AgF/KI applications can be considered acceptable approaches to reduce hypersensitivity in permanent molars affected by MIH both immediately and in long-term follow-up for schoolchildren based on caregivers’ assessments.

## 1. Introduction

Molar incisor hypomineralisation (MIH) is a well-known qualitative enamel defect that affects one to four first permanent molars (FPMs) and often includes the incisors [1]. A recent systematic review highlighted the substantial burden of MIH, revealing that 878 million individuals worldwide are currently affected, with 17.5 million new cases diagnosed annually [2]. The management of affected MIH molars, particularly in severe cases, presents a variety of clinical challenges. The compromised enamel is more likely to experience a post-eruptive breakdown, which increases the risk of carious lesion development and results in increased sensitivity and discomfort for patients during normal oral functions [3]. Hence, this results in increased extensive treatment needs, leading to a significant impact on a child’s quality of life and socio-psychological well-being [2]. Moreover, challenges in achieving effective anaesthesia and the higher rates of failure associated with adhesive restorations contribute to a lack of cooperation from children during treatment [4]. Based on the available literature, factors linked to MIH development during the pre- and perinatal periods include prematurity (defined as birth before 37 weeks), low birth weight, and cesarean delivery. Additionally, postnatal elements such as prevalent childhood illnesses, respiratory conditions, infections, and the use of antibiotics have also been correlated with MIH alongside a likely genetic predisposition [5].

The diagnosis of MIH teeth is typically made when the child is of early age, sometimes coinciding with their initial visit to the dentist [6]. Children with affected molars often complain of hypersensitivity, making it challenging for them to brush properly [7]. There are considerable differences in the morphological characteristics of enamel in teeth with MIH when compared to normal enamel. A previous SEM study demonstrated that the enamel surface of teeth affected by MIH is porous, reflecting the chemical, mechanical, and physical properties of the tissue [8]. Thus, this soft and porous hypomineralised enamel is prone to rapid breakdown after an eruption, especially under the pressure of chewing, leading to symptoms. Consequently, these children face a notably high risk of developing caries [9]. Based on the result of a previous clinical study, the prevalence of caries based on the dmft-index in the group of children affected by MIH was found to be 24.14%, in contrast to a prevalence of 11.18% observed in the group of children not affected by MIH [10]. The findings suggest that particular emphasis should be placed on the various pathological conditions frequently related to MIH molar due to the fact that children suffering from MIH demand more intensive preventive actions, as restorative treatments are reported to be up to ten times more common than in patients who do not have MIH, even when risk factors such as bad oral hygiene are absent [11,12].

Moreover, dental fear and anxiety (DFA) are commonly associated with MIH, leading to the suggestion that regular hypersensitivity, challenges in achieving local anaesthesia, and heightened treatment demands may induce DFA in individuals with MIH (Shields et al., 2024) [13]. Children with MIH often require more frequent treatments and may need retreatments. Based on the previous literature, by the age of nine, children with affected molars undergo treatment more often than those with sound molars without MIH [14]. Various minimally invasive techniques are recommended as preventative strategies against dental caries and hypersensitivity in hypomineralised molars [15]. Silver diamine fluoride (SDF) is a successful solution for active caries lesion stabilisation due to the combined remineralisation effects due to fluoride content and the antibacterial properties of silver. SDF is a non-invasive method that helps maintain the structure of the tooth, whether used alone as a chemotherapeutic option or in conjunction with a sealant such as glass-ionomer cement (GIC) known as SMART (silver modified atraumatic restorative technique) for caries and hypersensitivity treatment [16].

SDF offers significant and long-lasting relief from hypersensitivity by blocking dentinal tubules through the formation of fluorohydroxyapatite, which enhances the hardness and density of a mineral. In fact, the product was approved by the US Food and Drug Administration (FDA) in 2014 for treating dentinal hypersensitivity (MacLean, 2018) [17]. Several SDF products have been developed due to their increasing popularity. One such commercial product, Riva Star from SDI in Bayswater, Australia, contains 30–35% SDF and a saturated solution of potassium iodide (KI) for treating hypersensitive dentin [18]. KI can further decrease dentin permeability when applied following silver diamine fluoride [19]. However, glass-ionomer cement (GIC) sealant is suitable for placement in challenging clinical conditions as a temporary treatment when isolation is not ideal. They are less sensitive to technique, set quickly, and do not require additional steps like etching. This makes them a favourable option for situations where an MIH molar is not fully erupted, but sealing is necessary, especially in uncooperative, anxious children or patients with hypersensitive and painful molars [20]. The easy application process of glass-ionomer cement (GIC) sealant is considered a highly convenient choice for children, especially due to their additional benefits through fluoride release, making them useful for caries prevention [6].

Parental acceptance of silver diamine fluoride (SDF) application by parents for their children’s teeth is becoming increasingly important in the field of paediatric dentistry [21]. Although the clinical effectiveness of SDF is well-established, its overall acceptance as a valid and minimally invasive treatment option depends on how caregivers view its results and its alternatives. Based on previous studies, both parents and dentists share a common concern regarding the possibility of tooth discolouration following SDF application. The potential impact on the aesthetic look of a child’s teeth can understandably lead to concerns [22]. The issue of discolouration and dental anxiety is the primary consideration in parental acceptance when paediatric dentists are evaluating the possibility of recommending SDF treatment for young patients, especially those with MIH [23]. Therefore, understanding parental acceptance is crucial as it can either impede or enhance the successful implementation of SDF and GIC sealants in the treatment of paediatric dental cases.

The existing literature is notably deficient in addressing MIH treatment modalities required for the establishment of standardised diagnostic protocols, interdisciplinary teamwork, and a holistic approach to patient care. Advancements in research, education, and policy are vital for improving outcomes among paediatric patients. By addressing both clinical and psychosocial challenges to positively influence the overall quality of life for children impacted by MIH [24]. Previous studies have investigated the causes, symptoms, risk factors and various treatment methods for MIH. Nevertheless, there is a noticeable gap in understanding parental viewpoints and children’s acceptance of various treatment modalities for MIH hypersensitivity. This gap becomes particularly prominent when we consider the experiences and insights of parents whose children have undergone SDF treatment for MIH. The parental perspective is not simply an academic curiosity but a crucial factor that can significantly influence treatment outcomes, compliance, and the overall well-being of the child [25]. Previous studies involve assessing the acceptance of hypersensitive MIH treatment modalities based on results from a questionnaire filled out by the caregiver without the involvement of children themselves in the assessment [26]. Therefore, the aim of this prospective, comparative double-arm clinical study is to explore both parental satisfaction and children’s acceptance of silver fluoride/potassium iodide (AgF/KI) and GIC (glass-ionomer cement) sealant treatment modalities on hypersensitive permanent molars affected by MIH. Further aspects, including the potential for tooth discolouration, as well as the comfort levels of parents and children during the treatment process, were also examined.

## 2. Material and Methods

This study aimed to assess caregiver satisfaction and the children’s acceptance of AgF/KI (silver fluoride/potassium iodide; Riva Star Aqua; SDI Limited, Bayswater, VIC, Australia) and GIC (Ionostar + EasyGlaze, Lincoln, UK; VOCO GmbH, Cuxhaven, Germany) coverage for hypersensitive molars affected by MIH. Ethical approvals for both study arms were obtained from the Institutional Ethical Committee at the University of Greifswald, Germany, with the following numbers: Reg. Nr. BB 047/23 for GIC sealant and BB 066/22 for AgF/KI. Signed informed consent was obtained from all participating caregivers. Patients for the two study arms were recruited in the Paediatric Dentistry Department of the University Medicine of Greifswald in the time period from January 2021 to March 2024. The current study adheres to the STROBE guidelines for a non-randomised clinical study.

### 2.1. Selection of the Participants

Eligible participants were specifically identified as caregivers of children aged 6–11 years whose children had received a diagnosis of hypersensitive molar incisor hypomineralisation (MIH) in their permanent molars. The children were either new patients or recall patients with either newly erupted hypersensitive MIH molars with no history of previous desensitisation treatment or patients with persisting hypersensitivity. Children with persisting hypersensitivity were those who had a history of desensitising treatment (e.g., GIC sealant, AgF/KI), which was recorded based on their previous dental records. No participant assignment was involved in this study as both study arms were performed independently and in different time periods (January 2021–September 2022 for AgF/KI) and (May 2023–March 2024 for GIC sealant).

For the diagnosis, MIH molars were classified according to the MIH treatment need index (MIH-TNI) [26,27]. Healthy children with at least one MIH-affected molars showing hypersensitivity with a SCASS (Schiff Cold Air Sensitivity Scale) score of 2 or 3 with and without occlusal breakdowns categorised as MIH treatment need index (MIH-TNI) of 3 and 4a/b/c based on the Würzburger concept were included [27,28].

To qualify for participation, MIH molars with persistent hypersensitivity should have received no in-office sealing therapy or application of desensitising agent on the MIH molar within 1 month before participation in the study. The exclusion criteria of the study were children with systemic diseases who need special attention during their dental treatment, children presenting with acute pain or signs or symptoms to be treated, MIH molars with signs of irreversible pulpitis, and children who have allergies to any contents of the study materials. Two trained study dentists (RK and WA) examined potential children for inclusion. In this study, hypersensitive molars affected by MIH were selected based on their sensitivity to an air blast stimulus. The air was delivered via a standard dental unit air syringe for 1 s, positioned 1 cm from the tooth and directed perpendicularly to its occlusal surface. The fingers of the examiners were used to shield adjacent teeth. The SCASS scoring system was employed to assess the subjects’ reactions to the stimulus, with scores defined as follows: 0 for no response; 1 for no response but recognition of pain; 2 for a response involving movement away from the stimulus; and 3 for a response that includes movement away and a request to stop the stimulus, in accordance with SCASS criteria [28].

### 2.2. Intervention

This prospective, comparative clinical study involved two treatment modalities (glass-ionomer cement application vs. silver fluoride/potassium iodide) for included hypersensitive MIH tooth/teeth, as shown below in Table 1. Before sealing, the hypersensitive MIH teeth were cleaned with prophylactic paste for tooth-cleaning (Prophy paste, Henry Schein, Langen, Germany) using a bristle brush, and no local anaesthesia was required for both procedures. For the 12-week follow-up duration of the study, patients were advised to brush daily with fluoride toothpaste and weekly with a fluoride gel (12.500 ppm fluoride), which is recommended for children with high caries risk [29].

Regarding the AgF/KI application, specific post-application instructions were given to the caregiver, in which children were advised to refrain from drinking and eating for a period of 1 h. This precautionary measure was aimed at providing better retention to ensure the efficacy of the applied AgF.

Moreover, both desensitisation treatments (glass-ionomer cement application vs. silver fluoride/potassium iodide) were performed in the specialised paediatric university dental clinic either by paediatric specialists and post-graduate paediatric dentistry students, all of whom were briefed on instructions and manufacturer’s guidelines on carrying out the interventions based on the study protocols.

### 2.3. Sample Size Calculation

Sample size calculation was performed for the primary outcome of the study project, which focused on the clinical assessment of both treatment modalities in reducing hypersensitivity after 12 weeks, using the Schiff cold air sensitivity score (SCASS) and subjectively through the child using the Wong–Baker Facial Scale (WBFS) [30]. However, patients and parental acceptance are the focus of the present paper, which is determined to be the secondary outcome of the study projects mentioned above. This calculation was based on data from a previous study that aimed to assess the hypersensitivity relief of desensitising paste, showing a minimum significant difference of 1 in SCASS scores [31]. It involved a power analysis of 80% at a two-sided 0.05 significance level and resulted in a required enrolment of at least 22 participants to have at least 20 patients in the 12 weeks’ follow-up, considering a maximum risk of 10% drop-out. Therefore, a minimum sample size of 40 children (20 children for each arm of the study) was set.

### 2.4. Data Collection

In this study, patients’ acceptance toward both desensitisation treatments was assessed throughout the desensitisation procedures (AgF/KI and GIC) via two calibrated study dentists (RK and WA) according to the Frankl Behaviour Rating Scale (FBRS), which categorises the child’s behaviour in the dental clinic into four categories: rating 1 (definitely negative) to rating 4 (definitely positive) [32,33]. Parental satisfaction with AgF/KI and GIC sealant treatments for their children’s molars affected by MIH hypersensitivity was assessed using a 4-item questionnaire at two time points: immediately (15 min post application) and in the 12 weeks follow-up. The questionnaire employed a 5-point Likert scale, with responses ranging from “1: Strongly Disagree” to “5: Strongly Agree”. Furthermore, children’s assessments regarding MIH hypersensitivity relief from AgF/KI (Riva Star Aqua, SDI) and GIC sealant (Ionostar Plus and Easy Glaze, VOCO) after 12 weeks were performed via the assessment of the following statement: ‘I had less hypersensitivity on my MIH molars in the last 12 weeks’ with the 5-point Likert scale. Additional questions, such as willingness to pay EUR 20 for desensitisation treatment with AgF/KI due to no insurance coverage and overall assessment of AgF/KI taste, smell, and easiness of application, were considered. Lastly, caregivers’ insight on the most preferred treatment alternative to both study modalities (AgF/KI and GIC sealant) was addressed. Baseline characteristics such as the caregiver’s age, sex, and educational background were taken from the study questionnaire, while baseline characteristics of the child, such as age and sex, were taken from the patient’s digital dental records. The reflection on dental caries experience and oral health was based on dmft/DMFT and involved clinical examination of the child by one of the study’s dentists (RK or WA).

### 2.5. Data Analysis

The data were initially summarised descriptively, and the mean value and standard deviation were calculated. Means and standard deviations (SDs) were computed for all quantitative variables, whereas frequencies and percentages were determined for categorical variables.

To compare the two study groups, an unpaired sample *t*-test was utilised for normally distributed quantitative variables, including age, dmft (decayed, missing, filled teeth in primary dentition), DMFT (decayed, missing, filled teeth in permanent dentition) and the number of hypersensitive MIH molars per patients. The comparison of qualitative nominal variables, including the Frankl Behaviour Rating Scale (FBRS) between the two study groups, was conducted using Fisher’s exact tests.

Furthermore, the Mann–Whitney U test was used for the analysis of baseline and follow-up measurements that were not normally distributed. The intra- and inter-examiner reliability was calculated using Cohen’s Kappa test. The inter-examiner reliability for assessing the dental behaviour of the child via the Frankl Behaviour Rating Score (FBRS) between both calibrated study dentists (RK and WA) was 0.7 based on the Cohen-Kappa test, while the intra-examiner reliability weighted Kappa was 0.8. Significance was inferred at *p* < 0.05. Data were analysed using Microsoft Excel 2016 software for MacOS.

## 3. Results

This prospective, comparative double-arm clinical study involved 47 healthy children, consisting of 25 males and 22 females, with a mean age of 8.6 (±1.42) years and an average dmft of 7.73 (±3.16). The first arm involved 25 (53.2%) children, each having at least one MIH molar with and without enamel breakdown linked to high degree hypersensitivity with a mean SCASS sore of 2.56 (±0.50), which received a GIC sealant application. In contrast, the second arm (n = 22, 46.8%) received a desensitising treatment with AgF/KI. There were no statistically significant differences between the arms in terms of gender and age distributions, dmft/DMFT values, and mean baseline SCASS scores (*p* > 0.05). The baseline characteristics and caries profiles of the children are presented in Table 2.

Table 3 shows the baseline characteristics of the caregivers who participated in both arms of the study (n = 25; 53.2% for GIC vs. n = 22; 48.6% for AgF/KI). Caregiver education levels were classified into distinct categories based on the education of both fathers and mothers. Among the 47 caregivers, 36 (76.6%) of them were female, and the majority of them (n = 31, 66.0%) had a secondary school educational level and higher. No significant difference in age, sex and educational background were observed within both study groups (Table 3).

Initially, both study groups (AgF/KI and GIC) involved children with hypersensitive MIH molars (SCASS 2-3, as illustrated in Table 2) of high intensity. After 12 weeks of desensitisation treatment, most of these children (40 out of 44, 90.1%) reported experiencing less hypersensitivity on the affected MIH molars in the last 12 weeks compared to baseline. Only two children (n = 2, 4.4%) in both intervention groups (AgF/KI and GIC) did not show a reduction in MIH hypersensitivity after 12 weeks. A comparison of the two interventions revealed no statistically significant difference in their effectiveness for alleviating hypersensitivity pain, as assessed subjectively by the child (*p* = 0.87), as shown in Table 4.

Within the GIC sealant group, the majority of the children (n = 15, 60%) demonstrated definitely positive behaviours during the treatment (Table 5). In contrast, the AgF/KI group showed more positive behaviour compared to definitely positive behaviour (n = 18, 81.8% vs. n = 2, 9.1%). Interestingly, the differences in behaviour ratings within both study groups were statistically significant (*p* < 0.05).

Caregivers of children who participated in both study groups agreed to be satisfied with the procedures immediately (n = 19, 86.4% for AgF/KI and n = 19, 76.0% for GIC sealant application) and in 12 weeks of follow-up (n = 17, 85.0% for AgF/KI and n = 22, 91.6% for GIC sealant application). Regarding further recommendations of the corresponding treatments for other children (AgF/KI and GIC sealant), no significant difference was observed between AgF/KI and GIC sealant applications immediately and in 12 weeks of follow-up (*p* = 0.65). Furthermore, in terms of repeating the treatment (in case it is necessary), only two caregivers (8.2%) did not agree to repeat the GIC sealant application based on the 12-week follow-up assessment (Table 6).

Based on study results, a slight change (n = 6, 27.3% for 15 min post-application vs. n = 2, 10.0% in 12 weeks follow-up) can be observed in caregivers’ evaluation of the MIH molar appearance after AgF/KI application. Only one caregiver (n = 1, 4.1%) disagreed with the ‘The appearance of the MIH teeth is acceptable after the treatment’ statement within 12 weeks. Furthermore, there were no statistically significant differences in the perceptions of the caregivers regarding the effect of both treatment modalities (AgF/KI vs. GIC sealant) on MIH molars appearance immediately (15 min post-application) and in 12 weeks of follow-up (*p* > 0.05). Further distribution of the data is presented in Table 6.

In this study, caregivers’ preferences for alternative immediate (15 min post-application) desensitising treatment were also considered. Most caregivers whose children received immediate GIC application preferred desensitising paste (n = 10, 45.5%). In comparison, tooth restoration coverage (n = 15, 68.2%) was most preferred in the AgF/KI group, with a statistically significant difference between caregiver views on treatment alternatives for immediate MIH hypersensitivity treatment after receiving AgF/KI and GIC sealant application (*p* < 0.05), as shown in Table 7.

Regarding caregiver assessment of the ease of the AgF/KI application, 91.2% (n = 20) of caregivers either strongly agreed or agreed that the procedure was easy. Furthermore, the overall child perception during the AgF/KI application was found to be pleasant in more than half of the children, with 63.6% (n = 14) strongly agreeing or agreeing based on their caregivers’ assessment (Table 8). As for the acceptability of the AgF/KI taste by the children, only half of the caregivers reported that their children found the taste to be acceptable, with 50% strongly agreeing or agreeing, while 4.6% (n = 1) were neutral and 45.4% (n = 10) disagreed. Furthermore, all caregivers (n = 22, 100%) reported the AgF/KI application for MIH desensitisation to be fast, as shown in Table 8.

## 4. Discussion

This study adopted a prospective, cross-sectional design to assess both caregiver satisfaction and child acceptance of both silver fluoride/potassium iodide (AgF/KI) and GIC (glass-ionomer cement) sealant treatment modalities for hypersensitive permanent molars affected by molar incisor hypomineralisation (MIH). The study complied with STROBE guidelines for cross-sectional research, ensuring a structured and rigorous approach to both data collection and analysis. This study was performed in a paediatric dentistry department of a university dental hospital (University Medicine of Greifswald), which provides a wide range of dental treatment for children from families of all kinds of educational backgrounds, as shown in the baseline characteristics of caregivers (Table 2). Due to the fact that the study centre receives mainly referred children from primary dental care providers for further diagnosis and management of MIH, the patients reflect a larger region with a mixed profile. The primary focus of the present study was to investigate child acceptance and caregiver satisfaction regarding AgF/KI and GIC sealant application for hypersensitive molars impacted by MIH, a topic that has not been adequately addressed in the existing body of literature. To accomplish this focus, the Frankl Behaviour Rating Scale (FBRS) for children’s acceptance and a 5-point Likert scale questionnaire was selected as the most appropriate methods for evaluating caregiver views and child satisfaction levels [32,34].

Regarding hypersensitivity relief of AgF/KI vs. GIC sealant application after 12 weeks based on children’s perception, the study findings indicate that a significant proportion of children in both treatment groups (n = 40, 90.1%) experienced less hypersensitivity in the last 12 weeks. This aspect is crucial, as it addresses the subjective effect of the primary outcome of both treatment modalities (AgF/KI and GIC sealant application). The findings of a previous study revealed that the application of a sealing technique for hypersensitive molars contributes positively to the quality of life in children. Noteworthy enhancements in oral health-related quality of life (OHRQoL) were already apparent one week following treatment in those affected by hypersensitive MIH molars [35].

Furthermore, both AgF/KI and GIC sealant applications are suitable for use in challenging clinical scenarios as immediate desensitisation in children with dental anxiety. Their ease of application, quick setting time, and lack of need for intermediate procedures, such as etching, make them advantageous. This is particularly relevant in cases where a molar affected by MIH has not fully erupted or for non-cooperative or anxious children, as well as those experiencing hypersensitivity and pain in their molars, the straightforward application of GIC or AgF/KI is often more favourable and convenient [20]. In this study, children who received GIC applications showed significantly more definitely positive behaviour than the AgF/KI group (*p* < 0.05). This difference in behaviour scores might be due to the issue of taste acceptability is especially significant, considering that children usually have a low preference for bitter-tasting medications, and silver fluoride is known for its bitter metallic taste [36,37]. However, the result of another study reported high levels of acceptance for sealants in children aged between 3 and 16 years of age with improved overall patient acceptance of the dental visit and increased treatment experience [38].

The outcome of this study revealed high satisfaction levels (n = 17, 85.0% for GIC sealant vs. n = 22, 91.6% for AgF/KI) with both treatments after 12 weeks. This is similar to the finding of a previous study reporting a high parental satisfaction (77.5%) with the treatment of silver diamine fluoride (SDF) on hypersensitive MIH teeth [26]. It is important to highlight that the current literature is deficient in systematic studies that comprehensively evaluate caregiver satisfaction and acceptance of GIC application, especially concerning permanent molars impacted by MIH hypersensitivity. The present study shows an overall high acceptance of GIC application immediately after 15 min post application (76.0%) and in 12 weeks follow-up (91.6%). These results reflect the findings of a previous study that observed that individuals with MIH-affected teeth could experience substantial advantages from desensitising therapies (e.g., fluoride varnish or GIC), which can alleviate hypersensitivity, facilitate better oral hygiene practices, and reduce the number of dietary limitations [39].

A significant limitation of silver fluoride/potassium iodide (AgF/KI) is the permanent dark staining it causes on demineralised tooth surfaces, cavities, and the edges of restorations. However, the application of potassium iodide (KI) immediately following AgF treatment can substantially diminish the staining [40]. Studies have indicated that the degree of discolouration is reduced when using the combination of AgF and KI compared to AgF used in isolation [41]. However, in this study, more than half of the children (n = 14, 63.3% for immediate application vs. n = 12, 60.0% in 12 weeks follow-up) disagreed or totally disagreed that the appearance of the MIH worsened after AgF/KI application. This is somehow similar to the result of a similar study, where the majority of parents (58%) reported satisfaction with the appearance of their child’s MIH molars following the application of silver diamine fluoride (SDF) [26]. This is generally plausible as both treatment modalities (AgF/KI and GIC sealant) were placed on posterior permanent molars, which are aesthetically usually only partially visible while smiling, making the appearance of teeth after treatment a less concern. A previous study showed that caregivers were more receptive to silver diamine fluoride application for caries management when used in the posterior teeth rather than in the anterior teeth [42].

In addition to the desensitisation effect of AgF/KI, previous studies reported a significant effect of the material in arresting carious lesions in paediatric patients, especially in those with special health care needs and in clinical scenarios where pharmacological behaviour management strategies are either not selected by caregivers or are considered inappropriate [26,43]. However, the application of silver fluoride/potassium iodide (AgF/KI) neither restores the structure of the tooth nor enhances masticatory function or occlusion [15]. Therefore, it is advantageous to restore enamel breakdown or cavitated lesions on MIH teeth by placing a restoration or a sealant coverage that adheres to the tooth structure, through which the source of nourishment for any remaining microorganisms within the cavity is effectively eliminated [15]. The use of SMART (silver-modified atraumatic restorative technique) reflects the clinical advantages of combining two desensitisation modalities, which uses silver fluoride (AgF) to inhibit the cariogenic biofilm formation and reduce hypersensitivity, followed by glass-ionomer cement (GIC) coverage to enhance tissue remineralisation, reduce the biofilm formation, provide a cleansable surface, conceal any discolouration caused by SDF, thereby improving the overall aesthetics [44,45]. Based on the result of the recently published study, the effectiveness of SMART sealants was found to be effective in preventing enamel caries and significantly reducing hypersensitivity in MIH molars [46]. This might be relevant to the result of this study, in which caregivers of both study arms (AgF/KI and GIC sealant) completely agreed or agreed to recommend both treatment modalities for hypersensitivity treatment in MIH molars with no significant difference between the groups (*p* = 0.65). Due to the off-label use of AgF/KI for treatment of MIH hypersensitivity, that cannot be covered by the German health insurance system. Caregivers were asked if they were willing to pay EUR 20 for AgF/KI for the desensitising treatment, as shown in Table 6. In this study, a high proportion of caregivers (n = 22, 100.0% for immediate application vs. n = 17, 85% for 12 weeks follow-up) fully agreed to pay additionally for the material. The result of this study showed that the absence of a complex equipment requirement, along with the relatively low expense of the materials used in silver fluoride (AgF) treatment, contributes to its cost-effectiveness when compared to alternative methods. The current literature supports this assertion, indicating that AgF/KI is frequently employed as a minimally invasive topical treatment that necessitates only basic equipment. In contrast, the process of filling a carious lesion with glass-ionomer cement (GIC) demands at least portable equipment, thereby increasing overall costs [47,48].

Within paediatric dentistry, prioritising children’s comfort and cooperation during treatment is essential, and thus, a straightforward and minimally invasive application process can considerably affect the decisions of parents [49,50]. The present study showed that tooth restoration coverage (n = 15, 34.1% in AgF/KI arm) and desensitising paste (n = 13, 29.6% in GIC sealant arm) were classified as the most preferred alternative treatment options to both presented methods (AgF/KI and GIC sealant), as shown in Table 7. This might not be much different to the results of a recent questionnaire study targeting Swedish dentists, which demonstrated that composite resin restorations were predominantly chosen for restorative treatment of moderately affected first permanent molars. On the other hand, fluoride varnish application was preferred for treating mild MIH cases [51].

A significant number of caregivers expressed satisfaction regarding the application of AgF/KI on their child’s MIH molars, with 91% indicating agreement or strong agreement with the ease of application (Table 8). Furthermore, every caregiver (100%) acknowledged that the application process was fast. However, taste acceptability was identified as a challenge, with almost half of the caregivers (45.4%) disagreeing with the statement that the taste is acceptable. These outcomes can be supported by the main finding of a recent study, which reported all parents (100%) found that the application of silver fluoride for hypersensitive MIH teeth was simple and painless. In contrast, taste acceptability was problematic, as more than half of the parents (53%) considered it unacceptable [24]. It is important to highlight that the perception of taste is inherently subjective and can differ significantly across individuals and cultural settings. A recent systematic review indicated that various studies have found that children, in particular, may display diverse reactions to different flavours, shaped by factors including age, prior dental experiences, and cultural and parental influences [23]. The reason behind the high acceptance rate of AgF/KI, despite the existence of taste concerns, might be explained by the fact the clinical benefits of AgF/KI application outweigh the unpleasant taste factor. This can be confirmed by the main finding of a previous research, which indicates that parents and children frequently emphasise treatment outcomes more than the discomfort associated with taste [22].

A significant limitation of this study is the small sample size. The stringent inclusion criteria, which permitted only hypersensitive MIH molars with no history of prior desensitisation within 1 month before starting with the study, resulted in both study arms enrolling only 47 children over a span of three years. Another limitation is the absence of a negative control group (i.e., a group without GIC coverage or AgF/KI), which may have further influenced the interpretation of our results. However, we deemed it unethical to implement a negative control. Additional limitations include the subjective nature of hypersensitivity assessment and the awareness of participants regarding their involvement in a trial. Further recommendations involve investigation of potential influences, which might influence caregivers’ assessment via the 5-point Likert scale, such as providing additional questions regarding caregiver expectations and their assessment of overall study dentist communication. Previous studies suggested formulating items using negative phrasing to enhance the validity and reliability of the scale, reduce potential response biases, and ensure a more balanced evaluation of the caregivers’ response [52]. Additionally, future research comparing in-office desensitisation (e.g., AgF/KI and GIC coverage) to home-based desensitisation (e.g., daily brushing with fluoride toothpaste and weekly use of high-concentration fluoride gel) should be considered as an ethical approach for introducing control groups to better clarify treatment efficacy. It is important to note that compliance bias was unlikely to affect participant responses, as they were not personally connected to the investigator and were not provided with any incentives to take part in the study. Furthermore, children received the AgF/KI treatment for free, and the GIC application was covered by health insurance.

## 5. Conclusions

Based on key findings of the present study, caregiver satisfaction and children’s acceptance of GIC sealant and AgF/KI as both immediate and long-term desensitisation treatment options for permanent MIH molars are reported to be high. Both desensitisation options (AgF/KI and GIC sealant) are associated with positive dental behaviour and can be assessed as a positive approach to managing hypersensitivity associated with MIH. Additionally, this research enriches the existing literature in paediatric dentistry and highlights the necessity of considering parental opinions when devising treatment plans.

## Figures and Tables

**Table 1:** Treatment modalities of the study.

Treatment Modalities	Study Materials	Description
GIC Sealant	Ionostar Plus + Easy Glaze (VOCO GmbH, Germany)	The hypersensitive MIH tooth/teeth was/were sealed with a fast-setting radiopaque bulk-fill glass-ionomer filling material (Ionostar Plus, VOCO) after mixing the capsule for 10 s, followed by an application of a nano-filled, light-curing coating (Easy Glaze, VOCO) with a natural fluorescence for surface sealing cured for 30 s.
AgF + KI	Riva Star Aqua^®^ (SDI Limited, Australia)	Silver fluoride (AgF; Riva Star Aqua^®^, SDI Limited) was applied, followed by potassium iodide (KI) using a disposable microbrush applicator (MRG400, Henry Schein, Melville, NY, USA). The application process lasted one minute per tooth, thereby ensuring extensive coverage.

GIC: glass-ionomer cement; AgF: silver fluoride; KI: potassium iodine; MIH: molar–incisor incisor hypomineralisation.

**Table 2:** General baseline characteristic of the study sample (patient level; n = 47).

Patient Variable	Category	GIC Sealant Group (n = 25; 53.2%)	AgF/KI Group (n = 22; 46.8%)	Total	*p*-Value
Age (in years)	Mean (±SD)	8.6 (±1.85)	8.7 (±0.99)	8.6 (±1.42)	0.82 *
	Min–Max	6.2–11.7	6.2–10.3	6.2–11.7	
Sex	Males	11 (44.0%)	14 (63.6%)	25 (53.2%)	0.24 **
	Females	14 (56.0%)	8 (36.4%)	22 (46.8%)	
dmft	Mean (±SD)	2.46 (±3.06)	2.86 (±2.88)	2.66 (±2.97)	0.92 *
	Min–Max	0.00–9.00	0.00–5.00	0.00–9.00	
DMFT	Mean (±SD)	0.80 (±0.87)	1.64 (±1.40)	1.22 (±1.13)	0.60 *
	Min–Max	0.00–3.00	0.00–4.00	0.00–4.00	
SCASS	Mean (±SD)	2.56 (±0.50)	2.50 (±0.50)	2.53 (±0.50)	0.68 *
	Min–Max	2–3	2–3	2–3	
Number of hypersensitive MIH molars per patient	Mean (±SD)	1.72 (±1.02)	2.41 (±1.10)	2.07 (±1.06)	0.64 *
	Min–Max	1.00–4.00	1.00–4.00	1.00–4.00	

SD: standard deviation, Min: minimum, Max: maximum, * unpaired *t*-test, ** Fisher’s exact test.

**Table 3:** General baseline characteristic of the study sample (parental level; n = 47).

Patient Variable	Category	GIC Sealant Group (n = 25; 53.2%)	AgF/KI Group (n = 22; 46.8%)	Total (n = 47, 100%)	*p*-Value
Age (in years)	Mean (±SD)	39.8 ± 6.26	37.9 ± 4.20	38.5 ± 5.37	0.23 *
	Min–Max	27–52	30–45	27–52	
Sex	Females	20 (80.0%)	16 (72.7%)	36 (76.6%)	0.73 **
	Males	5 (20.0%)	6 (27.3%)	11 (23.4%)	
Educational background (Father)	≤Secondary school	8 (32.0%)	8 (36.3%)	16 (34.0%)	0.76 *
	>Secondary school	17 (68.0%)	14 (63.7%)	31 (66.0%)	
Educational background (Mother)	≤Secondary school	8 (32.0%)	8 (36.3%)	16 (34.0%)	0.76 *
	>Secondary school	17 (68.0%)	14 (63.7%)	31 (66.0%)	

SD: standard deviation, Min: minimum, Max: maximum, * unpaired *t*-test, ** Fisher’s exact test.

**Table 4:** Child response to questionnaire regarding MIH hypersensitivity relief of AgF/KI (Riva Star Aqua, SDI) and GIC sealant (Ionostar Plus and Easy Glaze, VOCO) after 12 weeks (n = 44; drop-out, n = 3).

Statement	Material	Fully Agree/Agree	Neither	Disagree/Totally Disagree	Total N (%)	*p* Value
		N (%)				
I had less hypersensitivity on my MIH molars in last 12 weeks	AgF/KI	18 (90.0%)	1 (5.0%)	1 (5.0%)	20 (100%)	
	GIC sealant group	22 (91.6%)	1 (4.2%)	1 (4.2%)	24 (100%)	
Total		40 (90.1%)	2 (4.4%)	2 (4.4%)	44 (100%)	0.87 *

* Mann–Whitney U test.

**Table 5:** Comparison of dental behaviour via Frankl Behaviour Rating Scale (FBRS) during both interventions in both study groups (n = 47).

Frankl Behaviour Rating Scale (FBRS)		Study Groups		Total N (%)	*p*-Value *
Characteristics	Score	GIC Sealant Group (n = 25; 53.2%)	AgF/KI Group (n = 22; 46.8%)	47 (100%)	
Definitely negative	1	-	-	-	
Negative	2	3 (12.0%)	2 (9.1%)	5 (10.6%)	
Positive	3	7 (28.0%)	18 (81.8%)	25 (53.2%)	
Definitely positive	4	15 (60.0%)	2 (9.1%)	17 (36.2%)	
Mean (±SD)		3.5 (±0.71)	3.0 (±0.43)	3.3 (±0.64)	<0.05

* Independent student *t*-test, statistically significant at *p*-value < 0.05.

**Table 6:** Parental responses to questionnaire immediately and after 12 weeks follow-up of AgF/KI (Riva Star Aqua, SDI) and GIC sealant (Ionostar Plus and Easy Glaze, VOCO) application on hypersensitive MIH molars (n = 44, drop-out; n = 3).

Statement	Material	Time Interval	Fully Agree/Agree	Neither	Disagree/Totally Disagree	Total N (%)	*p*-Value
			N (%)				
I am satisfied with the overall treatment.	AgF/KI	Immediately	19 (86.4%)	2 (9.1%)	1 (4.5%)	22 (100%)	0.97 *
		12 weeks	17 (85.0%)	3 (15.0%)	-	20 (100%)	
	GIC Sealant	Immediately	19 (76.0%)	5 (20.0%)	1 (4.0%)	25 (100%)	0.17 *
		12 weeks	22 (91.6%)	1 (4.2%)	1 (4.2%)	24 (100%)	
*p*-value **		0.44 **					
I would recommend this treatment to other children.	AgF/KI	Immediately	21 (95.5%)	1 (4.5%)	-	22 (100%)	0.51 *
		12 weeks	18 (90.0%)	2 (10.0%)	-	20 (100%)	
	GIC Sealant	Immediately	24 (96.0%)	1 (4.0%)	-	25 (100%)	0.52 *
		12 weeks	22 (91.6%)	-	2 (8.4%)	24 (100%)	
*p*-value *		0.65 **					
I would repeat the treatment if necessary.	AgF/KI	Immediately	21 (95.5%)	1 (4.5%)	-	22 (100%)	0.97 *
		12 weeks	19 (95.0%)	1 (4.5%)	-	20 (100%)	
	GIC Sealant	Immediately	24 (96.0%)	1 (4.0%)	-	25 (100%)	0.26 *
		12 weeks	22 (91.6%)	-	2 (8.4%)	24 (100%)	
*p*-value *		0.78 **					
The appearance of the MIH teeth worsened after the treatment.	AgF/KI	Immediately	6 (27.3%)	2 (9.1%)	14 (63.3%)	22 (100%)	0.56 *
		12 weeks	2 (10.0%)	6 (30.0%)	12 (60.0%)	20 (100%)	
The appearance of the MIH teeth is acceptable after the treatment.	GIC Sealant	Immediately	21 (84.0%)	4 (16.0%)	-	25 (100%)	0.63 *
		12 weeks	19 (79.2%)	4 (16.7%)	1 (4.1%)	24 (100%)	
I would pay EUR 20 for this material.	AgF/KI	Immediately	22 (100%)	-	-	22 (100%)	0.59 *
		12 weeks	17 (85.0%)	2 (10.0%)	1 (5.0%)	20 (100%)	
The hypersensitivity of MIH has a negative effect on OHRQoL	GIC Sealant	Immediately	14 (56.0%)	6 (24.0%)	5 (20.0%)	25 (100%)	0.29
		12 weeks	17 (70.8%)	4 (16.7%)	3 (12.5%)	24 (100%)	

* Mann–Whitney U test ** one-way ANOVA test.

**Table 7:** Comparison of parental view on treatment alternative for immediate MIH hypersensitivity treatment after receiving AgF/KI (Riva Star Aqua, SDI) and GIC sealant application (Ionostar Plus and Easy Glaze, VOCO) (n = 44: n = 3 non-responses in GIC group).

Alternative MIH Desensitising Treatment	Study Groups		Total N (%)	*p*-Value *
Treatment Option	GIC Sealant Group (n = 22; 50.0%)	AgF/KI Group (n = 22; 50.0%)	44 (100%)	
Tooth restoration coverage	-	15 (68.2%)	15 (34.1%)	<0.05
Desensitising paste	10 (45.5%)	3 (13.6%)	13 (29.6%)	
Stainless steel crown	5 (22.7%)	3 (13.6%)	8 (18.2%)	
Fluoride varnish	7 (31.8%)	1 (4.6%)	8 (18.1%)	

* Mann–Whitney U test statistically significant at *p*-value < 0.05.

**Table 8:** Parental responses to questionnaire regarding the immediate AgF/KI (Riva Star Aqua, SDI) application on hypersensitive MIH molars (n = 22).

Statement	Fully Agree/Agree	Neither	Disagree/Totally Disagree	Total N (%)
	N (%)			
The application was easy for my child.	20 (91.0%)	1 (4.5%)	1 (4.5%)	22 (100%)
The application was pleasant for my child.	14 (63.6%)	5 (22.8%)	3 (13.6%)	22 (100%)
The taste was acceptable for my child.	11 (50.0%)	1 (4.6%)	10 (45.4%)	22 (100%)
The smell was acceptable for my child.	14 (63.6%)	6 (27.3%)	2 (9.1%)	22 (100%)
The treatment was fast.	22 (100%)	-	-	22 (100%)

## Data Availability

The raw data supporting the conclusions of this article will be made available by the authors upon reasonable request. Basically, all data are presented in the article.
